# Short Term Real-Time Rolling Forecast of Urban River Water Levels Based on LSTM: A Case Study in Fuzhou City, China

**DOI:** 10.3390/ijerph18179287

**Published:** 2021-09-02

**Authors:** Yu Liu, Hao Wang, Wenwen Feng, Haocheng Huang

**Affiliations:** 1 Faculty of Architecture, Civil and Transportation Engineering, Beijing University of Technology, Beijing 100124, China; liuyu@emails.bjut.edu.cn; 2 School of Water and Environment, Chang’an University, Xi’an 710054, China; fww@chd.edu.cn; 3 School of Civil Engineering, Central South University, Changsha 410075, China; hhccsu@csu.edu.cn

**Keywords:** urban river management, water level forecasting, real-time rolling forecast, LSTM

## Abstract

Water level management is an important part of urban water system management. In flood season, the river should be controlled to ensure the ecological and landscape water level. In non-flood season, the water level should be lowered to ensure smooth drainage. In urban areas, the response of the river water level to rainfall and artificial regulation is relatively rapid and strong. Therefore, building a mathematical model to forecast the short-term trend of urban river water levels can provide a scientific basis for decision makers and is of great significance for the management of urban water systems. With a focus on the high uncertainty of urban river water level prediction, a real-time rolling forecast method for the short-term water levels of urban internal rivers and external rivers was constructed, based on long short-term memory (LSTM). Fuzhou City, China was used as the research area, and the forecast performance of LSTM was analyzed. The results confirm the feasibility of LSTM in real-time rolling forecasting of water levels. The absolute errors at different times in each forecast were compared, and the various characteristics and causes of the errors in the forecast process were analyzed. The forecast performance of LSTM under different rolling intervals and different forecast periods was compared, and the recommended values are provided as a reference for the construction of local operational forecast systems.

## 1. Introduction

Urban rivers are important parts of urban landscapes and drainage systems. In the non-flood season, rivers need to maintain water levels that meet the requirements of the landscape and ecology [1]. In flood season, as the outlet of drainage networks, rivers need to lower the water levels to ensure smooth drainage [2]. Through the construction of a mathematical model and real-time forecast system, the future river water regime can be forecasted, which can effectively provide a scientific basis for decision makers to specify a reasonable scheduling strategy [3,4,5].

In urban areas, due to the small volume of water storage and the high speed of runoff generation and confluence, the response of river water levels to rainfalls and water-diversion works is rapid and intense, and the flood control or ecological operation can be completed within a few hours [6,7]. Therefore, the forecast system should focus on short-term forecasting [8]. If the system can predict the trend of water level changes in the next 1–6 h, it will be enough to provide support for decision makers.

Traditional river water level simulations are usually based on the hydrology/hydrodynamics method, which can be forecast by solving the differential equations describing the whole runoff generation, confluence, and river flow [9,10]. This method describes the complete physical process with high precision and a clear explanation of physical mechanisms [11]. However, the data requirements are more stringent, and the basic data, such as river section shape, river length, and reservoir capacity characteristics, are required to establish the model. In addition, the rainfall, flow, and water level processes should be provided as the boundary conditions [9]. In real-time forecast systems, other models are often used to forecast the boundary conditions to extend the forecast period, which will lead to more computing resources and error accumulation [12].

To solve the above problems, the machine learning method has been applied to hydrological and hydrodynamic time series forecasts [13,14,15]. This kind of method can forecast future trends by exploring the temporal and spatial evolution of historical data [16]. The model usually takes the time series data of one or more time steps in the past (such as water level, rainfall, etc.) as the input, and the river water level of one or more time steps in the future as the output [17,18,19]. Of the many machine learning algorithms, such as the support vector machine (SVM), k-nearest neighbor (KNN), and linear discriminant analysis (LDA), the artificial neural network (ANN) has shown superior performance for hydrological time series data [20,21,22].

Long and short-term memory (LSTM) is a special recursive neural network [23]. It includes a concept of “control gates” to control the flow of information, and prevent the possible model perturbations caused by useless data, thus solving the problem of training long sequences and the gradient of recursive neural networks [24]. Therefore, compared to other neural networks, LSTM can make better use of the time-varying characteristics of time series data [24]. In recent years, many researchers have explored the application of LSTM in hydrological time series forecasting and have achieved good results [25,26]. Therefore, LSTM was adopted in this study.

In the process of river water dispatching, decision makers usually make corresponding decisions according to the current situation and future trends, which is a rolling process [27]. Therefore, the rolling forecast method can also be used in the operational application of river water level forecasting. With this method, the forecast system inputs the latest monitoring data according to the specified forecast frequency, and each simulation extends the given forecast period from the initial rolling time to the future [12]. At present, the LSTM method has been proven to be suitable for short-term water situation forecasting, but few studies have been carried out on the operational application of LSTM in the rolling forecast of river water levels. The frequency of the rolling forecast and the forecast period of each forecast are important parameters in a rolling forecast system. The impact of different rolling forecasting modes on forecasting performance remains to be discussed.

In this paper, we present an application case of LSTM in a real-time rolling forecast of river water levels in Fuzhou City. The time series of river water levels in Fuzhou city from 1 April to 1 October 2020 were collected and the forecast network was trained. Then, based on the trained network, the real-time forecast process of the whole test period was retrieved through the data that was obtained at each historical time point. The forecast performance of LSTM under different rolling intervals and different forecast periods was evaluated.

This paper is organized as follows: Section 2 presents the hydrological situation of the study area, the source of data, the principle of the model, the rolling forecast method, and the model performance evaluation method. In Section 3, the performance of LSTM in the rolling forecast of river water levels is presented. The absolute errors of the model at different forecast times are calculated and analyzed, and we compare the performance of the model for different rolling intervals and different forecast periods. In Section 4, the influence of different river sections on LSTM performance, the necessity of real-time rolling forecasting, and the recommended values of rolling intervals and forecast periods are discussed. At the same time, we point out the deficiency of the current form of forecasting systems and make suggestions for the future development of forecasting systems. Brief conclusions are given in Section 5.

## 2. Methods

### 2.1. Study Area and Data

Fuzhou is the capital of Fujian Province, a coastal city in Southeast China, at 119°17′18″ E and 26°04′08″ N. The study area is located in the north of Fuzhou City, surrounded by mountains in the east, west, and north, and adjacent to Minjiang River in the south. The river network is densely distributed in the area, with a drainage area of 165.4 km^2^ and a total length of 147.9 km. The tidal level of Minjiang River is between 0.5 m and 6.0 m, while the average elevation of some urban areas in the study area is only 5.5 m, which is lower than the highest tide level. Thus, a large number of sluices, pumps, and other projects have been built in the area to regulate the water level of the inland river. During periods of no rainfall, it is necessary to prevent the inland river from drying up with the tide receding as well as from pouring back with the tide rising. The average annual precipitation in the study area is 1394 mm, which is mainly concentrated between April and October, accounting for about 80% of the rainfall for the whole year. During the rainstorm period, the rainwater pipe network in the urban area is discharged into the inland river nearby and threatened by the mountain torrents in the north. If the tide of Minjiang River rises, the inland river cannot drain water, causing the rapid rise of the water levels of the inland river, hindering the drainage of the pipe network, and causing water to accumulate in the urban area, which has a great impact on the regional traffic and peoples’ lives. The prediction of water levels can effectively support the decision making of local river dispatching. Moreover, under such natural and artificial conditions, the change in water levels of urban rivers is complex but has internal laws, which is of high research value.

**Figure 1:**
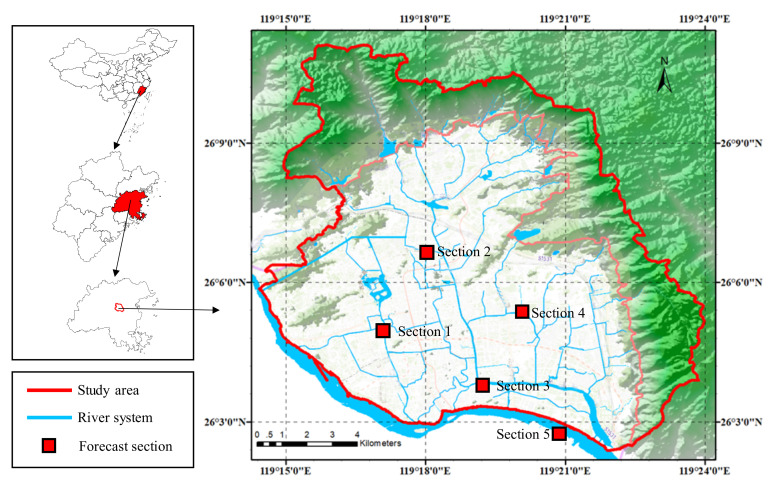
Distribution of the river system and river gauges in Fuzhou city.

The inland rivers and Minjiang River in the study area are all equipped with water level gauges. As shown in Figure 1, the selected water level gauges are located in five different river cross-sections: four of them are considered internal rivers and one is the external Minjiang River. Section 1 is located in the west of the basin, at the intersection of Baima and Xinxi Rivers. Section 2 is located in the middle reaches of Huqian river and Section 3 is located at the intersection of Jin’an and Guangminggang Rivers. Section 4 is located in the middle reaches of Fengban river and Section 5 is located in Minjiang River. Each section is equipped with an ultrasonic water level gauge, which is transmitted and stored in the database through IoT (Internet of Things) technology as input for the model.

### 2.2. Method and Implementation

#### 2.2.1. LSTM (Long Short-Term Memory)

LSTM (long short-term memory) is a kind of time RNN (recurrent neural network) that is suitable for processing time series data and which has been widely used in hydrological time series data forecasting [17]. LSTM is different from RNN in that there is an added “processor” in the algorithm to judge whether the information is useful or not. The structure of this processor is called a cell.

**Figure 2:**
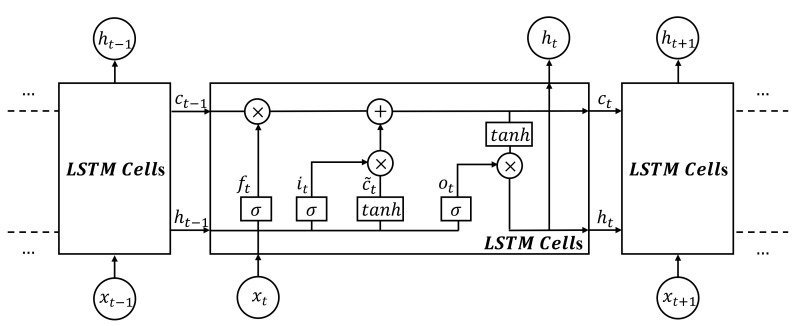
Neuron in the hidden layer of LSTM.

As shown in Figure 2, three gates, called the input gate, the forget gate, and the output gate are placed in an LSTM cell. When information enters the LSTM network, it is judged as to whether it is useful according to the rules. Only the information that conforms to the algorithm authentication will remain, and the inconsistent information will be forgotten through the forget gate. The calculation method for each gate is shown in Equations (1)–(6) [28].

Input gate:

(1) $$i_{t}=\sigma \left(W_{i}x_{t}+R_{i}h_{t-1}+b_{i}\right)$$

Forget gate:

(2) $$f_{t}=\sigma \left(W_{f}x_{t}+R_{f}h_{t-1}+b_{f}\right)$$

Output gate:

(3) $$o_{t}=\sigma \left(W_{o}x_{t}+R_{o}h_{t-1}+b_{o}\right)$$

Cell state:

(4) $${\tilde{c}}_{t}=tanh\left(W_{c}x_{t}+R_{c}h_{t-1}+b_{c}\right)$$

(5) $$c_{t}=f_{t}⊗c_{t-1}+i_{t}⊗{\tilde{c}}_{t}$$

Output vector:

(6) $$h_{t}=o_{t}⊗tanh\left(c_{t}\right)$$

where $x_{t}$ is the input vector and $i_{t}$, $f_{t}$, and $o_{t}$ are the input, forget, and output gates state vectors at time t. $c_{t}$ is the LSTM cell state vectors at time t. $h_{t}$ is the output gate of the cell state vector at time t. σ denotes the Sigmoid activation function. tanh denotes the hyperbolic tangent function. $W_{i}$, $W_{f}$, and $W_{o}$ denote the matrix of weights from the input, forget, and output gates to the input layer, respectively. Similarly $R_{i}$, $R_{f}$, and $R_{o}$ denote the matrix of weights from the input, forget, and output gates to the hidden layer, respectively. $W_{c}$ and $R_{c}$ are the matrix of weights of the LSTM cell. $b_{i}$, $b_{f}$, $b_{o}$, and $b_{c}$ denote the input, forget, and output gates, and the LSTM cell bias vectors. ⊗ represents the element-wise multiplication of two vectors.

#### 2.2.2. Real-Time Rolling Forecast Method and Implementation

**Figure 3:**
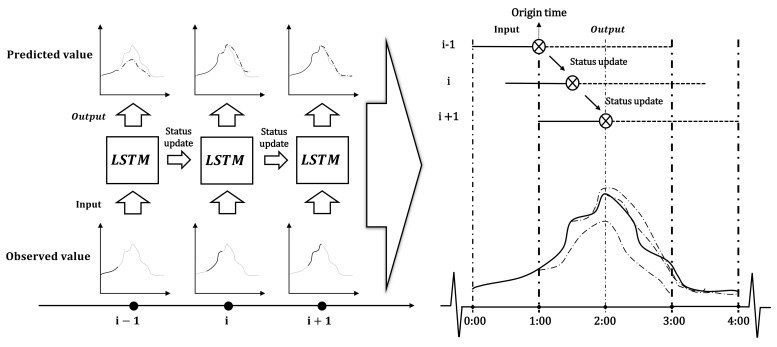
Schematic of the real-time forecast method based on LSTM.

Due to artificial control and natural factors, such as sluices, pumps, rainfall, and tides, the variations in river water levels are highly uncertain [29]. Therefore, it is necessary to consider the rolling forecast method [30,31,32,33]. In this study, the river section water levels were forecast by the rolling forecast method, as shown in Figure 3. At the beginning of each simulation, the most recent observed river water level data is used as the input, the unit state is updated, and the change trend of water level is forecast. The start time of each forecast is recorded as the origin time. Then, the interval between the two origin times determines the rolling interval, and the length of each output data determines the forecast period of each simulation.

The purpose of the forecast system is to forecast the water levels several steps in the future according to the water levels of several steps in the past as observed by IoT. The time resolution of the data collected by IoT is 10 min, so the time resolution of water levels in the forecast system is also 10 min. In this study, 12, 24, 36, and 48 steps were tested as inputs respectively. The results show that the model performed best when 36 steps were input. In order to make a fair comparison of the impact of the forecast period and rolling interval on the performance of the model, the inputs for each model were the water levels of the past 36 steps (6 h). The output was the water level process every 10 min for the following several hours. After confirming the input data and forecast goals, the structure of LSTM was optimized through trial-and-error procedures. Single-layer and double-layer LSTM and 50, 100, 150, 200, and 300 hidden units were tested to determine the best LSTM structure. The structure of LSTM was determined as single-layer LSTM with 200 hidden units.

The ADAM optimization algorithm was used to train the network [34]. ADAM is an algorithm for first-order gradient-based optimization of stochastic objective functions, which is straightforward to implement and is based on adaptive estimates of lower-order moments of the gradients. According to the recommended values in the references and repeated optimization tests, the values of the relevant training parameters were as follows: the gradient threshold was 1, the initial learning rate was 0.005, the drop period of learning rate was 125, the drop factor of learning rate was 0.2, and the maximum number of iterations was 125. The model and data processing program used in this study were implemented by MATLAB 2021a (Mathworks, Natick, MA, USA).

#### 2.2.3. Forecast Performance Evaluation Method

As shown on the right side of Figure 3, when the time interval between two forecasts is shorter than the length of each forecast period, the water level forecast sets from different origin times will be generated at the same time. To evaluate the performance of the forecast, the root mean square error (RMSE), the Nash efficiency coefficient (NSE), and the goodness of fit (R^2^) were used to evaluate the performance of the model. The calculation methods of RMSE, NSE, and R^2^ used in this study are shown in Equations (7)–(9), respectively.

(7) $$\mathrm{RMSE}=\sqrt{\frac{\sum_{i=1,j=1}^{n,m}{\left(Y_{i，j}^{obs}-Y_{i,j}^{sim}\right)}^{2}}{nm}}$$

RMSE is used to measure the deviation between the observed value and the true value. The closer the value is to 0, the smaller the error is.

(8) $$\mathrm{NSE}=1-\left[\frac{\sum_{i=1,j=1}^{n,m}{\left(Y_{i,j}^{obs}-Y_{i，j}^{sim}\right)}^{2}}{\sum_{i=1,j=1}^{n}{\left(Y_{i,j}^{obs}-Y_{obs}^{mean}\right)}^{2}}\right]$$

NSE varies from negative infinity to 1. If the NSE is close to 1, it means that the quality of the model is good and the model is reliable. If the NSE is close to 0, it means that the simulation results are close to the average of the observed values, that is, the overall results are reliable, but the process simulation error is large. If the NSE is far lower than 0, the model is not credible.

(9) $$R^{2}=\left[\frac{{\left(\sum_{i=1,j=1}^{n,m}\left(Y_{i,j}^{sim}-Y_{sim}^{mean}\right)\left(Y_{i,j}^{obs}-Y_{obs}^{mean}\right)\right)}^{2}}{\sum_{i=1,j=1}^{n,m}{\left(Y_{i,j}^{sim}-Y_{sim}^{mean}\right)}^{2}\sum_{i=1}^{n}{\left(Y_{i,j}^{obs}-Y_{obs}^{mean}\right)}^{2}}\right]$$

The R^2^ values range between 0 and 1, and an R^2^ value of 1 indicates a perfect correlation.

In the above-listed equations, i indicates different times, j indicates different simulations, n is the total number of time steps, m is the total number of simulations, $Y_{i，j}^{obs}$ is the observed value at time i in the jth simulation, $Y_{i，j}^{sim}$ is the simulated value at time i in the jth simulation, $Y_{obs}^{mean}$ is the observed average, and $Y_{sim}^{mean}$ is the simulated average.

## 3. Results

### 3.1. Data Sets for Training and Testing

In this study, the river water level observation data of each river section from 1 April to 1 October 2020 were collected. The observation intervals were 10 min, and a total of 26,353 records were collected. The data from 1 April to 1 August 2020 was used as the training period to train the model, using 17,569 records. The test period was from 1 August to 1 October, using 8784 records. The summary of the water level statistical data of each section is shown in Table 1. It can be seen that the statistical values of training period data and forecast period data are close, with high similarity.

### 3.2. The Performance of the LSTM Forecast Model in the Whole Test Period

**Figure 4:**
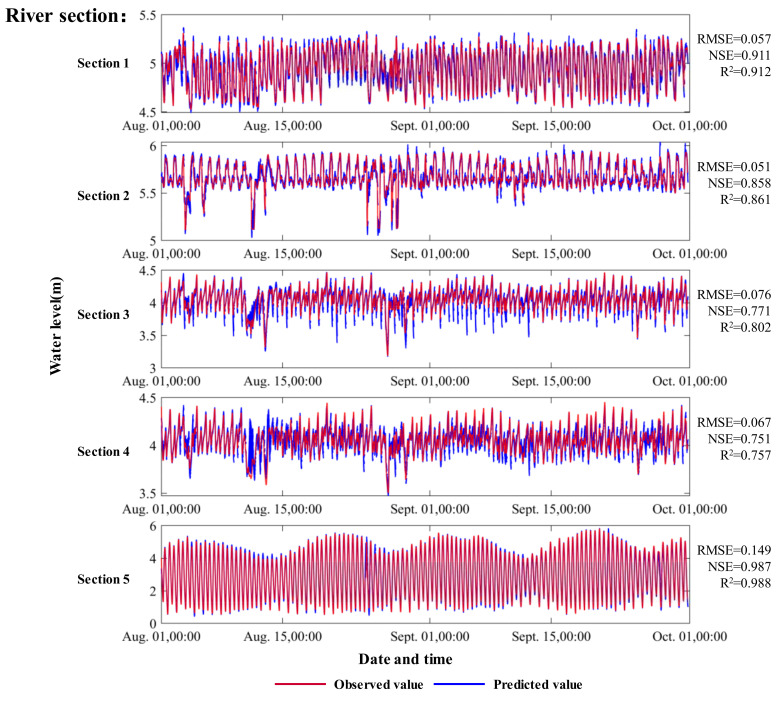
Comparison of observed and simulated water levels.

This section presents the evaluation of the overall performance of LSTM for the forecasting of water levels of urban rivers in the complete test period through a long series of case studies. In the whole test period, the single forecast time for a river section was less than 20 ms, which met the timeliness requirements of real-time prediction. The rolling interval was 30 min and the forecast period was 2 h (the proof that these were appropriate values is presented in Section 4.3), and the comparisons between the observed values and the simulated values are shown in Figure 4. It can be seen that, on the whole, the simulated values of the five sections fit well with the observed values.

Specifically, the five river sections have different variation rules. Among them, the bottom elevations of river Sections 1 and 2 are relatively high, and the water levels are mainly affected by the water diversion pump station and are between 4.5 m and 5.5 m, and 5.1 m and 5.8 m, respectively. The RMSE, NSE, and R^2^ of the two sections were 0.057, 0.911, and 0.912, and 0.051, 0.858, and 0.861, respectively. Sections 3 and 4 are located downstream of the whole basin. The bottom elevations of these sections are low and part of the tidal channel. The water level is mainly affected by the gate and tide level along the river and is between 3.3 m and 4.5 m. The RMSE, NSE, and R^2^ of the two sections were 0.076, 0.771, and 0.802, and 0.067, 0.751, and 0.757, respectively. Section 5 is located on Minjiang River, which is a natural river water level with no regulation of the sluice and pump. The water level is only affected by the tide, ranging from 0.4 m to 5.9 m. The RMSE, NSE, and R^2^ of Section 5 were 0.149, 0.987, and 0.988, respectively, which showed the best fitting performance.

### 3.3. Error Analysis of Different Simulation Time

**Figure 5:**
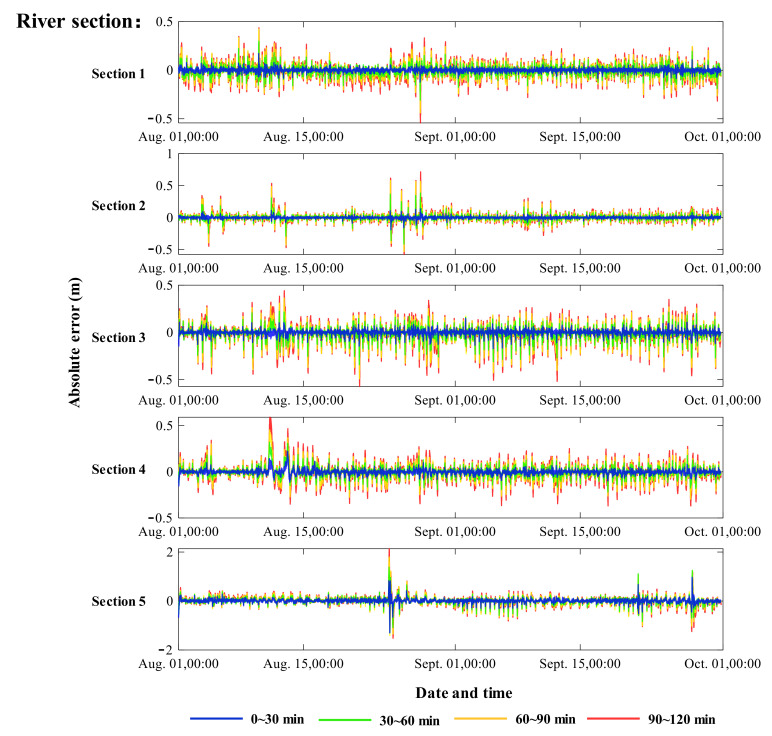
Absolute error between the observed and simulated water levels.

When the rolling interval is 30 min and the forecast period is 2 h, a set of four forecast results are generated at each time point, including the latest forecast, the forecast 30 min ago, the forecast 60 min ago, and the forecast 90 min ago. The absolute error between the forecast value and the observed value is obtained by subtracting the result and the observed value at each time, which is shown in Figure 5. The absolute error statistics are shown in Table 2. These results also represent the absolute error of each forecast result in different time periods, namely 0–30 min, 30–60 min, 60–90 min, and 90–120 min.

It can be seen in Figure 5 and Table 2 that the five river prediction sections have good prediction performance with a low error rate. In general, each simulation error increases with the increase of simulation time. The average errors of the five river sections in the next 0–30 min are 0.011 m, 0.017 m, 0.015 m, 0.015 m, and 0.046 m respectively, while the average errors in the next 90–120 min are 0.064 m, 0.080 m, 0.077 m, 0.070 m, and 0.128 m respectively, which are several times larger than the former.

The absolute error of Section 5 is obviously higher than that of the other four sections, which is due to the larger variation range of the water level. However, from the perspective of the change degree, the average error of Section 5 in the next 90–120 min is 2.81 times those of 0–30 min, which are 0.128 m and 0.046 m respectively, while the increase of the other four sections is more than 4 times.

### 3.4. Performance of LSTM in Different Forecast Periods and Rolling Intervals

**Figure 6:**
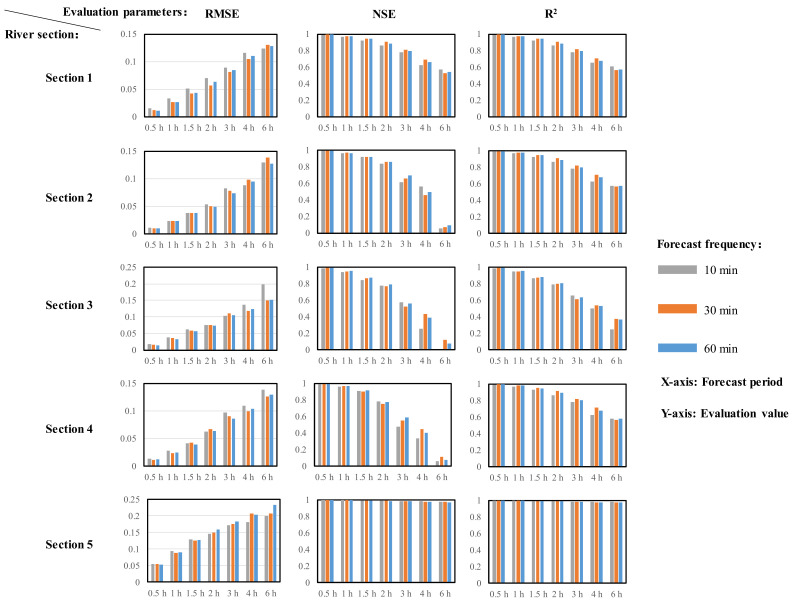
Model evaluation under different forecast periods and rolling intervals.

In order to further evaluate the performance of LSTM under different prediction frequencies and different prediction periods, the rolling intervals were 10 min, 30 min, and 60 min, and the prediction periods were 0.5 h, 1 h, 1.5 h, 2 h, 3 h, 4 h, and 6 h, respectively. The RMSE, NSE, and R^2^ under different conditions were calculated to evaluate the performance of the model, and the results are shown in Figure 6.

The forecast period has a great influence on the performance of the model. With an increase in the forecast period, the RMSE increased, and the NSE and R^2^ decreased, which made the prediction performance worse. When the prediction period was 0.5 h, the RMSE of the five prediction sections was between 0.01 and 0.05, while when the prediction period was 6 h, the RMSE increased to between 0.13 and 0.24. When the prediction period was 0.5 h, the NSEs of the five prediction sections were greater than 0.97, while when the prediction period was 6 h, the NSE of river Section 1 was reduced to 0.5, Sections 2, 3, and 4 were significantly reduced to below 0.2, and Section 5 was slightly reduced to 0.9. Similarly, when the prediction period was 0.5 h, the R^2^ of the five prediction sections was greater than 0.98, while when the prediction period was 6 h, the R^2^ of Sections 1, 2, 3, and 4 were significantly reduced to between 0.3 and 0.6, while the R^2^ of Section 5 was not significantly reduced to 0.9.

The rolling intervals have no obvious rule on the forecast performance of the model. Under the same forecast period, the performance of the model was similar under different rolling intervals.

## 4. Discussion

### 4.1. Impact of River Section Characteristics on LSTM Performance

LSTM had different forecast performance for the five selected river sections in this study. This is due to the different influence factors on water level changes in five river sections.

For internal rivers, due to the characteristics of hydraulic regulation engineering and narrow river channels, the water levels are greatly and rapidly affected by the gate/pump control, rainfall, and other factors. The water level change trend is highly uncertain. In a forecast, LSTM identifies the current and past water regime and predicts the water level change process in the next few hours. However, in a forecast, due to the changes of the above factors, the future water level trend also changes correspondingly, and the accumulated error increases rapidly with time. Therefore, the error of each forecast stage shows the characteristics shown in Section 3.3; with an increase in forecast time, the absolute error increases rapidly. It further leads to the result in Section 3.4, that is, with an increase of the forecast period, the forecast performance of LSTM on the internal river water level decreases rapidly.

In contrast, for external rivers, less hydraulic regulation engineering and wider channels cause the changes in water levels to be less affected by sluice/pump control, rainfall, and other factors. The changes in water level are mainly affected by the tide and follow its currents. Therefore, as shown in Section 3.3 and Section 3.4, the absolute error in each period of a forecast is relatively close, and the overall performance of the forecast will not decrease too much with an increase in the forecast period.

### 4.2. Necessity of Real-Time Rolling Forecast Method

In urban areas, where the changes in river water levels are highly uncertain, the action of gates, pumping stations, and short-term heavy rainfall will have a greater impact on the changes. Perhaps based on the information available at the moment, a forecast for Minjiang River was once accurate. However, with the passage of time, the engineering and natural conditions in the region have changed, resulting in inaccurate results in the later period. Although this moment is still within the coverage of the last forecast, the forecast results need to be updated according to the latest observed data [35].

When the method shown in Section 2.2.2 is applied to the rolling forecast of river water levels, at the origin time of each forecast, the latest observation that can be obtained is used as the input of LSTM to update the most accurate future forecast trend at this time. It can be seen from Section 3.3 that in each forecast, the error is the lowest at the beginning of each simulation and increases with time. At the beginning of a new forecast, the model will be corrected by the latest observations, which effectively prevents the error accumulation caused by long-term forecasting.

### 4.3. Value Recommendation of Rolling Interval and Forecast Period

With the increase in the forecast period, the uncertainty of the water level changes increases, so the performance of LSTM on river water level forecasting becomes worse. The rolling intervals did not affect the overall performance of LSTM. Because the performance of each forecast is only determined by the input data and water level change law at that time, the forecast performance of the model is relatively close under different forecast frequencies in the same forecast period. As can be seen from Section 3.3, the error of each prediction is the smallest at the beginning and increases with the passage of forecast time. In a past study, although the rolling prediction method was used, when the simulation results were updated, the part covered by the new simulation results was ignored [24]. The evaluation method in Section 2.2.3 considers the overall accuracy of each forecast. Even if the new prediction results have been updated, the accuracy of the past prediction is still considered. However, the rolling interval is still an important parameter in a rolling forecast. As analyzed above, each forecast result performs best in the first 30 min, and the forecast error increases as the forecast time goes on. Therefore, a shorter rolling interval means that the model can identify the latest trend of water level changes in time and take the latest forecast results as the basis of decision making to cover the previous forecast results. A longer rolling interval will make LSTM unable to update in time and obtain the latest trend of water levels, which will affect the timing of decision making. In addition, the rolling interval will greatly affect the computing resources. Therefore, it is of vital importance to choose an appropriate rolling frequency in the process of real-time rolling forecasting.

Due to the results of this case study, for the internal river, the value of the forecast period is recommended to be no more than 2 h; under such conditions, the NSE can be guaranteed to be higher than 0.8. The rolling interval is recommended to be no more than 30 min to ensure the timeliness of the updated forecast results. For external rivers, a longer forecast period and rolling interval can be selected because, as the results show, when the forecast period is 6 h, the model still performs well, as the NSE is higher than 0.9.

### 4.4. Future Development of the System

Hydraulic control engineering (such as pumps and gates) and natural conditions (such as rainfall) may have a significant impact on urban inland water levels [36]. The current form of this prediction system does not input these factors into the LSTM model. This is because the monitoring system of the gate and pump station in the study area is not complete. In addition, due to the rapid response of urban rivers to these factors, in order to extend the prediction period of river water levels, it is necessary to input the predicted values of these factors. Due to the transmission of error, this may lead to poor prediction performance. Further research is needed to discuss the related issues.

At present, the forecast system can provide decision makers with timely and accurate predictions of water level change trends, but it still needs decision makers to make the control plan for the gate and pump station. In the future, the forecast model may be combined with an optimization algorithm or decision tree to automatically recommend the optimal control plan for decision makers [37].

## 5. Conclusions

Based on the LSTM, a real-time rolling forecast method for urban inland river and outer river water levels was constructed. The data of the observed water levels of five river channels in Fuzhou City from April to October 2020 were collected. Based on the collected data, the model was trained and tested, the error of the model with different forecast times was analyzed, and the forecast performance of the model in different rolling intervals and different forecast periods was evaluated. The main conclusions are as follows:

(1) LSTM can effectively forecast the short-term trend of urban river water levels in the study area. Under the conditions of a rolling interval of 30 min and a forecast period of 2 h, the model performs well in the forecast of five sections. Its highest RMSE is only 0.149, and its lowest NSE and R^2^ are 0.751 and 0.757, respectively.
(2) The absolute error at the beginning of each forecast is the smallest, and the longer the forecast starts, the greater the absolute error is. Through the real-time rolling forecast method, the forecast water level is corrected to the observed value at the beginning of each simulation, which avoids the error accumulation of long-time simulations.
(3) The forecast period has a significant impact on the performance of the model. Among the five selected river sections in the study area, the forecast system can still perform well for the external river when the forecast period is 6 h, but for the internal river, the forecast performance will deteriorate rapidly when the forecast period is more than 3 h. The rolling interval will not affect the overall accuracy of the model, but it means updating the speed of the model results, which determines whether the model can update the water level trend in time.
(4) In this study, only the water level was considered as the model input. In further studies, with the improvement of the local monitoring system, hydraulic control engineering and natural conditions should be considered as added input factors for the model. In addition, the model and optimization algorithm can be combined to develop an intelligent decision-making system.

## Figures and Tables

**Table 1:** Statistics of observed water levels in the training period and the testing period.

Forecast River Section	Max Level (m)		Min Level (m)		Average Level (m)		Standard Deviation Level	
	Training	Testing	Training	Testing	Training	Testing	Training	Testing
Section 1	5.71	5.34	4.37	4.47	4.91	4.94	4.91	4.94
Section 2	6.02	5.95	5.09	5.09	5.64	5.67	5.64	5.67
Section 3	4.77	4.49	2.97	3.17	3.98	4.05	3.98	4.05
Section 4	4.80	4.48	3.15	3.50	4.01	4.06	4.01	4.06
Section 5	6.47	5.86	0.42	0.49	2.96	3.01	2.96	3.01

**Table 2:** Statistics of absolute error of water levels in different forecast times.

Forecast River Section	Absolute Error (0~30 min)/m		Absolute Error (30~60 min)/m		Absolute Error (60~90 min)/m		Absolute Error (90~120 min)/m	
	Max	Mean	Max	Mean	Max	Mean	Max	Mean
Section 1	0.172	0.011	0.308	0.028	0.438	0.047	0.543	0.064
Section 2	0.166	0.017	0.319	0.036	0.458	0.058	0.599	0.080
Section 3	0.170	0.015	0.341	0.035	0.491	0.058	0.571	0.077
Section 4	0.224	0.015	0.321	0.032	0.454	0.052	0.592	0.070
Section 5	1.299	0.046	1.378	0.076	1.799	0.103	2.137	0.128

## Data Availability

The data presented in this study are available on request from the corresponding author. The data are not publicly available due to confidentiality.
